# Beverage consumption in patients with metabolic syndrome and its association with non-alcoholic fatty liver disease: a cross-sectional study

**DOI:** 10.3389/fnut.2024.1257969

**Published:** 2024-01-22

**Authors:** Chayanis Kositamongkol, Sorawis Ngaohirunpat, Supawit Samchusri, Thanet Chaisathaphol, Weerachai Srivanichakorn, Chaiwat Washirasaksiri, Chonticha Auesomwang, Tullaya Sitasuwan, Rungsima Tinmanee, Naruemit Sayabovorn, Phunchai Charatcharoenwitthaya, Pochamana Phisalprapa

**Affiliations:** ^1^Division of Ambulatory Medicine, Department of Medicine, Faculty of Medicine Siriraj Hospital, Mahidol University, Bangkok, Thailand; ^2^Department of Clinical Epidemiology, Faculty of Medicine, Thammasat University, Pathum Thani, Thailand; ^3^Faculty of Medicine Siriraj Hospital, Mahidol University, Bangkok, Thailand; ^4^Division of Gastroenterology, Department of Medicine, Faculty of Medicine Siriraj Hospital, Mahidol University, Bangkok, Thailand

**Keywords:** caffeine, cocoa, coffee, fibrosis, metabolic syndrome, non-alcoholic fatty liver disease, steatosis, tea

## Abstract

**Introduction:**

Previous research has examined the association between coffee and tea consumption and non-alcoholic fatty liver disease (NAFLD). Preclinical studies have indicated the potential hepatoprotective properties of cocoa/chocolate. However, clinical research on the consumption of cocoa/chocolate and soft drinks and their relation to NAFLD, particularly among individuals with metabolic syndrome, is limited. This study primarily aimed to assess the association between beverage consumption and NAFLD in these patients.

**Methods:**

This cross-sectional study enrolled adult patients with metabolic syndrome visited the Medicine Outpatient Department at Siriraj Hospital, Thailand, from November 2011 to January 2013. The exclusion criteria were secondary causes of hepatic steatosis, such as excessive alcohol use, viral hepatitis, or drug-induced hepatitis. Participants completed a 23-item self-administered questionnaire covering their beverage consumption habits, including type, frequency, volume, duration, and additives in drinks, namely, coffee, tea, cocoa/chocolate, and soft drinks. To ensure accurate responses, these questionnaires were supplemented by face-to-face interviews. Ultrasonography was employed early in the methodology to diagnose NAFLD. Univariable analyses were used to compare the beverage consumption behaviors of participants with and without NAFLD. Multivariable logistic regression was used to adjust for potential confounders, including total beverage energy intake, age, anthropometric data, laboratory results, and comorbidities.

**Results:**

This study included 505 patients with metabolic syndrome. Of these, 341 (67.5%, 95%CI: 63.2–71.6%) were diagnosed with NAFLD. The consumption rates of coffee, cocoa/chocolate, and soft drinks were similar between the two groups. However, tea consumption was significantly more common in patients with NAFLD (68.3% vs. 51.8%, *p* < 0.001). The groups had no significant differences in caffeine intake or total energy intake from beverages. Notably, daily intake of three or more cups of coffee was correlated with a reduced prevalence of NAFLD, with an adjusted odds ratio of 0.35 (95%CI: 0.14–0.89).

**Conclusion:**

This study revealed that patients with metabolic syndrome, irrespective of NAFLD status, exhibited similar patterns of beverage consumption. While no definitive associations were identified between the intake of coffee, tea, cocoa/chocolate, or soft drinks and NAFLD, a notable exception was observed. A higher consumption of coffee (≥3 cups daily) was associated with a lower prevalence of NAFLD.

## 1 Introduction

Non-alcoholic fatty liver disease (NAFLD) has emerged as a significant global health concern affecting countless individuals. This disease is characterized by excessive fat accumulation in the liver, which accounts for more than 5% of the hepatic mass. It occurs in people who have little or no alcohol consumption and who lack identifiable secondary causes (1). Metabolic syndrome, which affects approximately 1 billion people worldwide (2), is closely linked with NAFLD. Metabolic syndrome includes various disorders, such as type 2 diabetes mellitus, hypertension, insulin resistance, and dyslipidemia. Managing NAFLD primarily involves lifestyle changes. These changes include following a nutritious diet, pursuing weight loss (essential for both lean and non-lean individuals with NAFLD), and increasing physical activity (3, 4). The United States Food and Drug Administration has not approved pharmacological treatments targeting NAFLD.

Recent guidelines from the American Association for the Study of Liver Diseases (5) highlight the beneficial impact of coffee on NAFLD and liver fibrosis, irrespective of caffeine content. Studies indicate potential mortality benefits associated with coffee consumption (6, 7). Coffee consumption is linked to reduced levels of liver enzymes, such as gamma-glutamyl transpeptidase, serum alkaline phosphatase, and alanine aminotransferase (ALT). Coffee consumption has also been shown to be effective at mitigating hepatic steatosis and fibrosis and reducing the risk of cirrhosis and hepatocellular carcinoma (8). Separately, green tea may have protective effects against liver fibrosis and NAFLD. These benefits are attributed to metabolic regulation, antioxidative activity, anti-inflammatory, and antifibrotic effects of green tea (9–11).

Moreover, preclinical studies have shown promising hepatoprotective effects of cocoa. These are attributed to the antioxidative stress and anti-inflammatory properties of components of cocoa such as caffeine, chlorogenic acids, diterpenes (e.g., cafestol and kahweol), theobromine, and polyphenols (12). Specifically, cocoa flavanols have been found to enhance insulin sensitivity, while dark chocolate consumption has been associated with a decrease in aspartate aminotransferase (AST) levels in patients with NAFLD, suggesting its potential therapeutic value (13, 14). Furthermore, polyphenols in cocoa have been reported to improve endothelial function in patients with non-alcoholic steatohepatitis (15). However, the exact mechanisms by which these beverages impact the progression of NAFLD remain unclear.

While food is the primary energy source, beverages also contribute significantly to daily energy intake. Commonly consumed non-alcoholic beverages such as tea, cocoa/chocolate, and soft drinks are often ingested with added sweeteners. Despite the availability of calorie-free alternatives, sugar remains the predominant sweetener, making these beverages a notable energy source. Calorie intake has been associated with body weight, suggesting that beverage consumption may have different effects on liver fat accumulation in individuals. However, the relationships between beverages other than coffee (such as tea, cocoa/chocolate, and soft drinks) and NAFLD and liver fibrosis have not been determined (16). The existing evidence is mainly derived from preclinical and pilot studies, with limited research conducted in humans, particularly individuals with metabolic syndrome. Therefore, our study explored the association between beverage consumption and NAFLD in patients with metabolic syndrome.

## 2 Materials and methods

### 2.1 Participants and ethics approval

This cross-sectional study was conducted at Siriraj Hospital from November 2011 to January 2013. The participants were male and female adults aged older than 18 years who were diagnosed with metabolic syndrome. Participants were selected randomly through their medical records at the internal medicine outpatient department. The exclusion criteria applied prior to participation were weekly ethanol consumption exceeding 21 drinks for males and 14 drinks for females and secondary causes of hepatic steatosis, such as significant alcohol consumption, viral hepatitis, or drug-induced liver conditions. Of the initially identified individuals, 505 met the eligibility criteria and consented to participate. These patients completed a questionnaire; underwent an interview, a physical examination, and blood tests; and were subjected to upper abdomen ultrasonography and/or transient elastography.

#### 2.1.1 Diagnosis of metabolic syndrome

Patients were prescreened for metabolic syndrome through their medical records. The diagnoses of metabolic syndrome were based on the National Cholesterol Education Program’s Adult Treatment Panel III (NCEP ATP III, 2001) and its 2004 modification. The core diagnostic criterion was central obesity (defined as a waist circumference of at least 90 cm in Asian men or 80 cm in Asian women). Additionally, a patient had to meet at least two of the following conditions: low high-density lipoprotein cholesterol (HDL-c) levels (<40 mg/dL in men, <50 mg/dL in women); plasma triglyceride levels ≥150 mg/dL or treatment for this condition; fasting plasma glucose levels ≥100 mg/dL or treatment for elevated glucose; and blood pressure > 130/85 mmHg or treatment for hypertension (17, 18).

#### 2.1.2 Ethics approval

The Siriraj Institutional Review Board authorized the research protocol (approval number: 529/2011). Written consent was obtained from each participant before they entered the study. This process ensured that they understood the purpose and procedures of the study and voluntarily agreed to participate.

### 2.2 Measurements

Participants in this study were asked to complete a questionnaire about their beverage consumption. The questionnaire included images of drinks commonly consumed in the Thai market and comprised 23 questions about the participants’ current drinking behaviors: beverage types, frequency, duration, volume, and additives in coffee, tea, cocoa/chocolate, and soft drinks (Supplementary material S1). For caffeine and energy intake calculations, nutritional information from product labels was used (Supplementary material S1 and Supplementary Table S1). When participants could not identify the beverage brand, standard values from the United States Department of Agriculture were applied.

Standardized methods were used for upper abdomen ultrasonography and/or transient elastography. Demographic data, medical history, blood pressure, and physical measurements (weight, height, waist circumference, and hip circumference) were recorded. Body mass index (kg/m^2^) was calculated using height and weight data obtained during the physical examination. Venous blood samples were collected after overnight fasting. All the data were collected within ±1 month of the ultrasonography date. Most patients underwent transient elastography within 1 month following ultrasonography.

### 2.3 Exposure: coffee, tea, cocoa/chocolate, and soft drinks

In our analysis, the primary exposure data—comprising the consumption of coffee, tea, cocoa/chocolate, and soft drinks—were derived from self-administered questionnaires and confirmed through individual interviews. Participants provided comprehensive details about their beverage consumption habits, including the type or brand consumed, the volume consumed daily and weekly, and any sweeteners or additives used. This methodology was uniformly applied across all beverage types, ensuring consistency in the data collection. Our data analyses then focused on quantifying the total consumption, caffeine content, and caloric intake of each beverage utilizing the nutritional information provided on product labels.

### 2.4 Outcomes: NAFLD and advanced liver fibrosis

Participants underwent standardized ultrasonography to assess the presence and severity of hepatic steatosis. Hepatic echogenicity was quantified using the “bright liver score” to evaluate steatosis severity. Two radiologists independently assigned scores to classify participants based on their bright liver scores, with any discrepancies resolved through consensus. A score of 0 denoted the absence of NAFLD, while scores from 1 to 3 indicated its presence. Furthermore, a subset of participants underwent transient elastography (FibroScan; Echosens, Paris, France) for hepatic stiffness measurement, with stiffness values exceeding 8 kPa indicating advanced liver fibrosis.

### 2.5 Statistical analysis

Descriptive statistics were used to analyze and report demographic information. Categorical variables are presented as frequencies and percentages. Continuous variables following a normal distribution are expressed as means ± standard deviations, and non-normally distributed data are reported as medians (interquartile ranges [IQRs]). Independent *t* tests and Mann–Whitney *U* tests were used to compare continuous variables between groups, while Fisher’s exact test was used to assess proportional differences. These univariable analyses examined the baseline characteristics and beverage consumption patterns of individuals in the NAFLD and non-NAFLD cohorts. Multivariable logistic regression models were used to calculate adjusted odds ratios for NAFLD prevalence, correlating daily beverage intake with that of participants with lower consumption (less than 1 cup per day). Adjustments were made for total daily energy intake from beverages; age; waist circumference; the AST-to-ALT ratio; the presence of impaired fasting glucose or diabetes mellitus; and serum albumin, triglyceride, and HDL-c levels. value of *p* <0.05 indicated statistical significance. All analyses were performed using Stata Statistical Software, release 15.1 (StataCorp LLC, College Station, TX, USA).

## 3 Results

### 3.1 Baseline characteristics

In this cohort study involving 505 patients with metabolic syndrome, the mean age was 61 ± 11 years, with females constituting 54% of the cohort. The mean waist circumference was 93 ± 11 cm, and the mean body mass index was 27.1 ± 4.5 kg/m^2^. A high prevalence of comorbidities was observed: dyslipidemia, 95%; hypertension, 90%; diabetes mellitus, 50%; and impaired fasting plasma glucose, 31%. Laboratory assessments revealed a mean glucose level of 123 ± 41 mg/dL, a mean glycated hemoglobin (HbA1c) level of 6.6% ± 1.2%, a median triglyceride level of 120 mg/dL (IQR, 88–168), a mean HDL-c level of 54 ± 14 mg/dL, and a mean low-density lipoprotein cholesterol level of 99 ± 33 mg/dL. The mean AST and ALT levels were 25 ± 12 U/L and 27 ± 20 U/L, respectively, with 85% of patients displaying ALT levels less than 40 U/L. The data, including medications used, are detailed in Table 1.

**Table 1:** Baseline and clinical characteristics of patients with and without non-alcoholic fatty liver disease.

**Patient characteristic**	**Mean ± SD (*n* = 505)**	**Non-NAFLD (*n* = 164)**	**NAFLD (*n* = 341)**	**Value of *p***
		**Mean ± SD**	**Mean ± SD**	
Age (years)	61 ± 11	64.1 ± 10.1	59.5 ± 10.9	<0.001
Male sex, *n* (%)	230 (46)	79 (48.1)	151 (44.3)	0.411
Weight (kg)	69.9 ± 13.8	65.2 ± 12.1	72.1 ± 14.0	<0.001
Height (cm)	160.4 ± 8.3	159.8 ± 8.8	160.7 ± 8.1	0.290
Body mass index (kg/m^2^)	27.1 ± 4.5	25.4 ± 4.0	27.9 ± 4.6	<0.001
Waist circumference (cm)	93 ± 11	89.1 ± 10.6	95.0 ± 10.3	<0.001
Hip circumference (cm)	100 ± 9	98.1 ± 8.4	101.2 ± 8.5	<0.001
Waist-to-hip ratio	0.9 ± 0.1	0.91 ± 0.08	0.94 ± 0.07	<0.001
Systolic blood pressure (mmHg)	132 ± 15	130.7 ± 14.2	133.0 ± 14.6	0.094
Diastolic blood pressure (mmHg)	79 ± 11	76.5 ± 10.9	80.7 ± 11.2	<0.001
Smoking, *n* (%)	41 (8)	21 (12.8)	20 (5.9)	0.007
Underlying disease, *n* (%)				
Impaired fasting plasma glucose	157 (31)	59 (36.0)	98 (28.7)	0.100
Diabetes mellitus	250 (50)	59 (36.0)	191 (56.0)	<0.001
Hypertension	454 (90)	145 (88.4)	309 (90.6)	0.442
Dyslipidemia	480 (95)	156 (95.1)	324 (95.0)	0.958
Laboratory data				
Aspartate transaminase (U/L)	24.9 ± 12.1	22.2 ± 9.5	26.1 ± 13.0	<0.001
Alanine transaminase (U/L), median (IQR)	21.0 (16.0, 31.0)	17.0 (13.0, 22.0)	24.0 (17.0, 36.0)	<0.001
AST/ALT ratio	1.1 ± 0.4	1.3 ± 0.4	1.0 ± 0.4	<0.001
Alkaline phosphatase (U/L)	71.6 ± 24.4	73.6 ± 27.4	70.7 ± 22.7	0.207
Total bilirubin (mg/dL)	0.5 ± 0.2	0.5 ± 0.2	0.5 ± 0.2	0.881
Direct bilirubin (mg/dL)	0.2 ± 0.1	0.2 ± 0.1	0.2 ± 0.1	0.495
Albumin (g/dL)	4.4 ± 0.3	4.3 ± 0.3	4.5 ± 0.3	<0.001
Globulin (g/dL)	3.3 ± 0.4	3.3 ± 0.4	3.3 ± 0.4	0.899
GGT (U/L), median (IQR)	29.5 (21.0, 49.0)	23.0 (18.0, 32.0)	35.0 (23.0, 57.0)	<0.001
FPG (mg/dL)	123 ± 41	112.9 ± 34.2	127.9 ± 43.2	<0.001
HbA1c (%)	6.6 ± 1.2	6.3 ± 1.1	6.8 ± 1.2	<0.001
Total cholesterol (mg/dL)	180 ± 37	179.7 ± 39.1	180.2 ± 36.6	0.872
Triglyceride (mg/dL), median (IQR)	120 (88, 168)	101.5 (75.5, 138.0)	131.0 (97.0, 177.0)	<0.001
HDL-c (mg/dL)	54 ± 14	57.5 ± 16.8	51.6 ± 12.5	<0.001
LDL-c (mg/dL)	99 ± 33	99.3 ± 33.3	98.9 ± 32.6	0.911
Hemoglobin (g/dL)	13.3 ± 1.5	13.0 ± 1.6	13.4 ± 1.5	0.001
Platelet count (× 10^9^/L)	258.3 ± 68.4	249.0 ± 66.9	262.9 ± 68.7	0.033
Medication data, *n* (%)				
Metformin	193 (38.2)	34 (20.7)	159 (46.6)	<0.001
Sulfonylureas	115 (22.8)	20 (12.2)	95 (27.9)	<0.001
Pioglitazone	25 (5.0)	6 (3.7)	19 (5.6)	0.511
Insulin	33 (6.5)	11 (6.7)	22 (6.5)	1.000
DPP-4 inhibitors	10 (2.0)	1 (0.6)	9 (2.6)	0.178
Acarbose	4 (0.8)	0	4 (1.2)	0.309
ACEIs	175 (34.7)	65 (39.6)	110 (32.3)	0.111
Angiotensin receptor blockers	149 (29.5)	36 (22.0)	113 (33.1)	0.012
Calcium channel blockers	209 (41.4)	71 (43.3)	138 (40.5)	0.564
Alpha blockers	68 (13.5)	27 (16.5)	41 (12.0)	0.210
Beta blockers	122 (24.2)	49 (29.9)	73 (21.4)	0.045
Diuretics	53 (10.5)	17 (10.4)	36 (10.6)	1.000
HMG-CoA reductase inhibitors	418 (82.8)	147 (89.6)	271 (79.5)	0.005
Fibrates	38 (7.5)	8 (4.9)	30 (8.8)	0.149
Ezetimibe	11 (2.2)	4 (2.4)	7 (2.1)	0.754
Vitamin E	24 (4.8)	4 (2.4)	20 (5.9)	0.118
Vitamin B complex	35 (6.9)	9 (5.5)	26 (7.6)	0.456
Aspirin	199 (39.4)	75 (45.7)	124 (36.4)	0.052

ACEIs, angiotensin-converting enzyme inhibitors; DPP-4, dipeptidyl peptidase-4; HDL-c, high-density lipoprotein cholesterol; HMG-CoA, hydroxy-methyl-glutaryl-coenzyme A; IQR, interquartile range; LDL-c, low-density lipoprotein cholesterol; NAFLD, non-alcoholic fatty liver disease; SD, standard deviation.

Patients with NAFLD had a younger mean age than did those without NAFLD (59.5 ± 10.9 vs. 64.1 ± 10.1 years; *p* < 0.001) and a greater mean body mass index and waist circumference. The prevalence of diabetes mellitus was markedly greater in the NAFLD group (56% vs. 36%; *p* < 0.001). Additionally, these patients exhibited elevated AST (26.1 ± 13.0 vs. 22.2 ± 9.5 U/L; *p* < 0.001) and ALT levels, with medians of 24.0 U/L (IQR, 17.0–36.0) versus 17.0 U/L (IQR, 13.0–22.0), respectively; *p* < 0.001. Conversely, patients without NAFLD exhibited better management of metabolic syndrome risk factors. This was indicated by lower levels for fasting plasma glucose (112.9 ± 34.2 vs. 127.9 ± 43.2 mg/dL; *p* < 0.001), HbA1c (6.3% ± 1.1% vs. 6.8% ± 1.2%; *p* < 0.001), and triglycerides (with a median of 101.5 mg/dL (IQR, 75.5–138.0) vs. 131.0 mg/dL (IQR, 97.0–177.0); *p* < 0.001), but a higher HDL-c level (57.5 ± 16.8 vs. 51.6 ± 12.5 mg/dL; *p* < 0.001). The medications used corresponded with their comorbidities. These findings are summarized in Table 1.

### 3.2 NAFLD and beverage consumption

This study provided several insights into the relationship between NAFLD and coffee, tea, cocoa/chocolate, and soft drink consumption.

The investigation of coffee consumption revealed no significant differences between the NAFLD and non-NAFLD groups in terms of both the proportion of coffee drinkers and the quantity consumed. Specifically, for participants without NAFLD, the median caffeine intake from coffee was 57.00 mg/day (IQR, 1.60–70.93), while it was 40.57 mg/day (IQR, 3.80–57.00; *p* = 0.366) for patients with NAFLD. Additionally, the median caloric contribution from coffee was similar between groups, with participants without NAFLD consuming 5.0 kcal/day (IQR, 0.7–59.8) and patients with NAFLD consuming 5.0 kcal/day (IQR, 1.4–37.5; *p* = 0.564). Hence, our analysis revealed no significant associations. Specifically, there were no links between overall coffee consumption, the amount of caffeine intake, and the energy derived from coffee in patients, regardless of their NAFLD status.

Conversely, tea consumption patterns differed notably. A greater proportion of tea consumption was observed in the NAFLD group (68.3%) than in the non-NAFLD group (51.8%; *p* < 0.001). The median caffeine intake from tea was substantially lower in participants without NAFLD (0.28 mg/day; IQR, 0–5.87) than in those with NAFLD (2.23 mg/day; IQR, 0–11.20; *p* = 0.002). Moreover, patients with NAFLD obtained significantly more energy from tea (3.44 kcal/day; IQR, 0–25.02) than did their non-NAFLD counterparts (0.07 kcal/day; IQR, 0–10.67; *p* < 0.001). Furthermore, our detailed analysis revealed that participants with NAFLD were more inclined to consume sweetened tea than were those without NAFLD (44.0% vs. 30.5%; *p* < 0.004), and they tended to add greater quantities of sugar to their homemade tea beverages.

In this study, the consumption patterns of cocoa/chocolate were analyzed in patients with and without NAFLD. The median caffeine intake from cocoa/chocolate in both cohorts was negligible at 0 mg/day (IQR, 0–0). Energy consumption from cocoa/chocolate varied slightly, with a median intake of 0 kcal/day (IQR, 0–10.51) in the non-NAFLD group and 1.67 kcal/day (IQR, 0–11.02; *p* = 0.447) in the NAFLD group. However, these differences in caffeine and energy intake were not statistically significant.

Soft drink consumption was prevalent in more than half of the participants in the study cohort, yet no notable differences were observed between patients with and without NAFLD. The daily intake of soft drinks was comparable across both groups. In terms of caffeine intake from soft drinks, individuals without NAFLD consumed a median of 0.46 mg/day (IQR, 0–3.59), while those with NAFLD consumed 0.82 mg/day (IQR, 0–3.69; *p* = 0.661). The median energy intake from soft drinks was 3.74 kcal/day (IQR, 0–20.90) in the non-NAFLD group and 4.52 kcal/day (IQR, 0–16.04) in the NAFLD group, with a value of *p* of 0.596, indicating no significant difference.

Overall, the aggregate caffeine consumption from all beverage types did not significantly differ between patients with and without NAFLD. The two groups exhibited a median caffeine intake of approximately 57 mg/day (57.00 mg/day [IQR, 14.80–93.83] for participants with NAFLD and 57.86 mg/day [IQR, 10.83–114.00] for those without NAFLD). Similarly, the energy intake from all beverages was analogous in both cohorts, with median values of 60.38 kcal/day (IQR, 20.74–141.13) for patients with NAFLD and 56.86 kcal/day (IQR, 20.07–140.79) for those without NAFLD (*p* = 0.829). These findings are detailed in Table 2.

**Table 2:** Comparison of drinking behavior between patients with and without non-alcoholic fatty liver disease.

**Drinking behavior**	**Non-NAFLD (*n* = 164)**	**NAFLD (*n* = 341)**	**Value of *p***
	**Mean ± SD**	**Mean ± SD**	
Coffee consumption, *n* (%)	129 (78.7)	275 (80.7)	0.635
Consuming black coffee, *n* (%)	82 (50.0)	175 (51.3)	0.849
Volume of coffee (ml/day)	250 (215, 250)	250 (250, 250)	0.831
Caffeine from coffee (mg/day)	57.0 (1.6, 70.9)	40.6 (3.8, 57.0)	0.366
Energy from coffee (kcal/day)	5.0 (0.7, 59.8)	5.0 (1.4, 37.5)	0.564
Tea consumption, *n* (%)	85 (51.8)	233 (68.3)	<0.001
Consuming black tea, *n* (%)	37 (22.6)	75 (22.0)	0.909
Volume of tea (ml/day)	250 (0, 250)	250 (0, 250)	0.003
Caffeine from tea (mg/day)	0.3 (0, 5.87)	2.2 (0, 11.2)	0.002
Energy from tea (kcal/day)	0.1 (0, 10.7)	3.4 (0, 25.0)	<0.001
Cocoa/chocolate consumption, *n* (%)	77 (47.0)	177 (51.9)	0.342
Volume of cocoa/chocolate (ml/day)	0 (0, 250)	250, (0, 250)	0.232
Caffeine from cocoa/chocolate (mg/day)	0 (0, 0)	0 (0, 0)	0.044
Energy from cocoa/chocolate (kcal/day)	0 (0, 10.5)	1.67 (0, 11.0)	0.447
Soft drink consumption, *n* (%)	98 (59.8)	201 (58.9)	0.923
Volume of soft drink (ml/day)	250 (0, 250)	250 (0, 250)	0.974
Caffeine from soft drink (mg/day)	0.5 (0, 3.6)	0.8 (0, 3.7)	0.661
Energy from soft drink (kcal/day)	3.7 (0, 20.9)	4.5 (0, 16.0)	0.596
Total caffeine from all beverages (mg/day)	57.9 (10.8, 114.0)	57.0 (14.8, 93.8)	0.604
Total energy from all beverages (kcal/day)	56.9 (20.1, 140.8)	60.4 (20.7, 141.1)	0.829

NAFLD, non-alcoholic fatty liver disease.

### 3.3 Advanced liver fibrosis and its associated factors

Within the cohort of patients diagnosed with NAFLD and advanced liver fibrosis, defined by liver stiffness measurements >8 kPa, no significant differences in age or sex were noted. Patients with advanced liver fibrosis exhibited elevated liver enzyme levels, with AST levels of 42.6 ± 23.8 U/L versus 24.8 ± 10.6 U/L in those without advanced liver fibrosis (*p* < 0.001). The alanine aminotransferase levels in the advanced liver fibrosis group were 44.0 U/L (IQR, 25.0–59.0) versus 23.0 U/L (IQR, 18.0–34.0) in the non-advanced liver fibrosis group (*p* < 0.001), and the gamma-glutamyl transferase levels were 59.0 U/L (IQR, 37.0–115.0) versus 33.0 U/L (IQR, 22.0–57.0) (*p* < 0.001). In contrast, patients without advanced liver fibrosis had better control of metabolic syndrome risk factors, as evidenced by lower HbA1c levels (6.6% ± 1.0% vs. 7.0% ± 1.1%; *p* = 0.014) and smaller waist circumferences (93.3 ± 9.4 cm vs. 97.2 ± 10.6 cm; *p* = 0.018), as detailed in Table 3.

**Table 3:** Comparison of clinical characteristics between non-alcoholic fatty liver disease patients with and without advanced liver fibrosis.

**Patient characteristic**	**No advanced liver fibrosis (*n* = 235)**	**Advanced liver fibrosis (*n* = 39)**	**Value of *p***
	**Mean ± SD**	**Mean ± SD**	
Age (years)	58.8 ± 10.1	61.8 ± 13.9	0.101
Male sex, *n* (%)	105 (46.7)	22 (56.4)	0.225
Weight (kg)	70.3 ± 12.1	74.5 ± 16.0	0.056
Height (cm)	160.6 ± 7.9	161.7 ± 9.8	0.414
Body mass index (kg/m^2^)	27.2 ± 3.9	28.3 ± 4.1	0.122
Waist circumference (cm)	93.3 ± 9.4	97.2 ± 10.6	0.018
Hip circumference (cm)	100.0 ± 8.0	100.9 ± 8.2	0.527
Waist-to-hip ratio	0.93 ± 0.07	0.96 ± 0.07	0.009
Systolic blood pressure (mmHg)	132.5 ± 14.4	133.6 ± 14.6	0.683
Diastolic blood pressure (mmHg)	80.5 ± 10.6	79.6 ± 12.5	0.636
Smoking, *n* (%)	11 (4.7)	7 (18.0)	0.007
Underlying disease, *n* (%)			
Impair fasting plasma glucose	72 (30.6)	7 (18.0)	0.048
Diabetes mellitus	117 (49.8)	28 (71.8)	0.134
Hypertension	212 (90.2)	39 (100.0)	0.054
Dyslipidemia	223 (94.9)	38 (97.4)	0.701
Aspartate transaminase (U/L)	24.8 ± 10.6	42.6 ± 23.8	<0.001
Alanine transaminase (U/L), median (IQR)	23.0 (18.0, 34.0)	44.0 (25.0, 59.0)	<0.001
AST/ALT ratio	0.97 ± 0.33	1.04 ± 0.44	0.244
Alkaline phosphatase (U/L)	70.9 ± 19.7	88.4 ± 45.0	<0.001
Total bilirubin (mg/dL)	0.54 ± 0.25	0.57 ± 0.23	0.503
Direct bilirubin (mg/dL)	0.21 ± 0.10	0.25 ± 0.11	0.047
Albumin (g/dL)	4.4 ± 0.3	4.4 ± 0.4	0.786
Globulin (g/dL)	3.3 ± 0.4	3.4 ± 0.4	0.115
GGT (U/L), median (IQR)	33.0 (22.0, 57.0)	59.0 (37.0, 115.0)	<0.001
FPG (mg/dL)	121.9 ± 39.7	130.1 ± 32.1	0.223
HbA1c (%)	6.6 ± 1.0	7.0 ± 1.1	0.014
Total cholesterol (mg/dL)	182.5 ± 35.8	177.0 ± 31.5	0.364
Triglyceride (mg/dL), median (IQR)	131.0 (97.0, 183.0)	128.0 (103.0, 154.0)	0.812
HDL-c (mg/dL)	52.3 ± 12.7	49.6 ± 11.8	0.227
LDL-c (mg/dL)	100.1 ± 32.9	94.8 ± 27.6	0.338
Hemoglobin (g/dL)	13.5 ± 1.4	13.4 ± 1.6	0.615
Platelet count (×10^9^/L)	266.7 ± 68.5	233.1 ± 64.1	0.005

HDL-c, high-density lipoprotein cholesterol; IQR, interquartile range; LDL-c, low-density lipoprotein cholesterol; SD, standard deviation.

### 3.4 Advanced liver fibrosis and beverage consumption

The study revealed no significant correlations between daily beverage consumption (volume, energy intake, or caffeine intake) and the presence of advanced liver fibrosis, as shown in Table 4.

**Table 4:** Comparison of drinking behavior between non-alcoholic fatty liver disease patients with and without advanced liver fibrosis.

**Drinking behavior**	**No advanced liver fibrosis (*n* = 235)**	**Advanced liver fibrosis (*n* = 39)**	**Value of *p***
	**Mean ± SD**	**Mean ± SD**	
Coffee consumption	190 (80.9)	28 (71.8)	0.202
Drinking black coffee, *n* (%)	123 (52.3)	19 (48.7)	0.731
Volume of coffee (ml/day)	250 (250, 250)	250 (0, 250)	0.330
Caffeine from coffee (mg/day)	40.7 (3.8, 57.0)	57.0 (0, 57.0)	0.851
Energy from coffee (kcal/day)	5.5 (0.7, 33.3)	5.0 (0, 41.9)	0.672
Tea consumption	160 (68.1)	21 (53.9)	0.100
Drinking black tea, *n* (%)	53 (22.6)	9 (23.1)	1.000
Volume of tea (ml/day)	250 (0, 250)	250 (0, 250)	0.108
Caffeine from tea (mg/day)	2.6 (0, 12.0)	1.2 (0, 12.0)	0.433
Energy from tea (kcal/day)	4.0 (0, 29.39)	2.0 (0, 37.25)	0.380
Cocoa/chocolate consumption	123 (52.3)	19 (48.7)	0.731
Volume of cocoa/chocolate (ml/day)	250 (0, 250)	0 (0, 250)	0.719
Caffeine from cocoa/chocolate (mg/day)	0 (0, 0)	0 (0, 0)	0.187
Energy from cocoa/chocolate (kcal/day)	1.7 (0, 12.6)	0 (0.0, 34.1)	0.627
Soft drink consumption	144 (61.3)	24 (61.5)	1.000
Volume of soft drink (ml/day)	250 (0, 250)	250 (0, 250)	0.863
Caffeine from soft drink (mg/day)	0.9 (0, 3.85)	0.9 (0, 2.2)	0.351
Energy from soft drink (kcal/day)	5.5 (0, 18.1)	7.2 (0, 17.4)	0.887
Total caffeine from all beverages (mg/day)	57.0 (15.9, 96.3)	57.9 (13.7, 114.0)	0.815
Total energy from all beverages (kcal/day)	62.3 (24.7, 150.0)	75.6 (10.5, 156.4)	0.793

### 3.5 Prevalence of NAFLD and advanced liver fibrosis in relation to beverage consumption

Table 5 reveals that high coffee consumption (≥3 cups daily) was inversely related to NAFLD prevalence, with an adjusted odds ratio of 0.35 (95% CI: 0.14–0.89). However, the consumption of tea, cocoa/chocolate, or soft drinks was not significantly associated with NAFLD or advanced liver fibrosis prevalence (data not shown).

**Table 5:** Prevalence of non-alcoholic fatty liver disease in relation to beverage consumption.

**Cups/day**	***n***	**NAFLD prevalence n (%)**	Adjusted odds ratio* (95% CI)
Coffee			
0–<1	235	166 (70.6)	Ref
1–2	239	158 (66.1)	0.77 (0.49, 1.20)
≥3	31	17 (54.8)	**0.35 (0.14, 0.89)**
Tea			
0–<1	412	273 (66.3)	Ref
1–2	80	61 (76.3)	1.57 (0.84, 2.92)
≥3	13	7 (53.9)	0.82 (0.21, 3.20)
Cocoa/chocolate			
0–<1	447	302 (67.6)	Ref
1–2	55	37 (67.3)	1.38 (0.69, 2.78)
≥3	3	2 (66.7)	2.81 (0.23, 34.03)
Soft drinks			
0–<1	450	301 (66.9)	Ref
1–2	51	38 (74.5)	2.05 (0.96, 4.41)
≥3	4	2 (50.0)	0.35 (0.04, 3.00)

CI, confidence interval; NAFLD, non-alcoholic fatty liver disease.* The adjusted factors were total energy per day from beverages, age, waist circumference, aspartate aminotransferase-to-alanine aminotransferase ratio, impaired fasting glucose or diabetes mellitus, albumin, triglycerides, and high-density lipoprotein cholesterol. Bold value refers to the statistically significant value (*p* <0.05).

## 4 Discussion

Our study revealed that the baseline characteristics of patients with NAFLD were associated with poor control of metabolic syndrome indicators (HbA1c, fasting plasma glucose, triglycerides, body mass index, and waist circumference). Interestingly, a substantial intake of coffee, quantified as three or more cups daily, was negatively correlated with the prevalence of NAFLD. However, this study revealed no significant associations between NAFLD or advanced liver fibrosis and consumption habits related to cocoa/chocolate and soft drink consumption. The factors investigated were current drinking status, beverage type, and the caffeine content and caloric contribution of each beverage.

Notably, energy intake from tea consumption was significantly greater among patients with NAFLD than among those without NAFLD. Nevertheless, analysis of the total energy intake from all beverage types revealed a non-significant disparity between the two groups. This observation is particularly noteworthy in the context of Thailand’s beverage culture, where high-calorie tea drinks, such as sweetened tea and milk tea, are prevalent.

The significantly greater total caloric intake from tea among patients with NAFLD could be attributed to the prevalent consumption of high-calorie Thai beverages. These include Thai tea and sugar-enhanced infused teas, which are highly popular and typically contain substantial amounts of sugar, condensed milk, milk, and whipped cream per serving. A detailed analysis revealed differences in consumption patterns. Participants with NAFLD exhibited a greater propensity for consuming high-calorie teas, such as Thai tea and sugar-added instant tea, than did those without NAFLD (44.0% vs. 30.5%, *p* < 0.004). Additionally, they tended to add more sugar to their homemade tea. In contrast, participants without NAFLD tended to consume unsweetened tea, which resulted in lower caloric intake from tea.

Approximately half of our study participants were diagnosed with diabetes mellitus. This comorbidity could potentially influence their beverage preferences by leading them to avoid sugar-enhanced drinks. Nonetheless, our analysis revealed a comparable proportion of individuals consuming sugar-containing beverages among those with and without diabetes mellitus (approximately 60% among coffee consumers and 30% among tea drinkers). Additionally, we endeavored to perform subgroup analyses to elucidate the differential impact of sugar-added and non-sugar-added beverage consumption on NAFLD prevalence. Regrettably, these analyses yielded no statistically significant results, potentially due to confounding factors that were not identified or controlled for in our study.

However, the literature consistently underscores the relationship between the intake of sugar-rich beverages and an elevated risk of NAFLD. Specifically, fructose, a prevalent sugar component, has been implicated as a key factor in the development of NAFLD, and numerous studies have linked its consumption to increased inflammation and fibrosis (19–21). Additionally, a 2023 study by Sun et al. substantiated a positive correlation between the consumption of sugar-sweetened beverages and the fatty liver index, which represents hepatic steatosis severity (22).

Multiple hypotheses posit that caffeinated beverages may decelerate the progression of chronic liver disease. Preclinical investigations have demonstrated that caffeine impedes liver steatosis progression by restoring redox balance, suppressing TGF-β expression, and inhibiting hepatic stellate cell activity, whereas polyphenols exhibit antioxidative effects (23–25). Conversely, certain human studies have not established a significant correlation between coffee consumption and liver steatosis. This may be due to the impracticality of consuming the high doses (6–7 cups of coffee daily) necessary to mitigate liver steatosis in humans (26). The relationship between coffee intake and NAFLD prevalence remains inconclusive, with various studies presenting disparate results. The National Health and Nutrition Examination Survey indicated a marginal decrease in the prevalence of NAFLD with increased coffee consumption, but the effect was minimal (27). Furthermore, a meta-analysis encompassing six studies with 20,064 participants did not reveal a significant association (12).

Additional longitudinal research is imperative to elucidate the nexus between coffee and NAFLD. The 2023 guidelines from the American Association for the Study of Liver Diseases suggest that coffee consumption, irrespective of caffeine content, could be advantageous. They recommend the intake of three or more cups of coffee daily, barring contraindications, based on epidemiological research and a meta-analysis associating such consumption with a reduced risk of NAFLD and liver fibrosis (5, 28–31). Our findings align with these guidelines, indicating an association between the consumption of three or more cups of coffee daily and a lower prevalence of NAFLD. However, our data also demonstrated that total caffeine intake does not significantly vary between individuals with and without NAFLD.

Our investigation has multiple strengths, particularly the exhaustive collation of data regarding diverse beverage types. This analysis included detailed consumption frequency and volume metrics and included calculations of the caffeine content for each beverage category. The primary basis of our analysis was grounded in self-reported data, wherein participants meticulously detailed their consumption patterns. This included specifying the type or brand of beverages, the quantity consumed daily, the weekly frequency, and any additional additives. The data were acquired through a meticulously designed self-administered questionnaire and were subsequently corroborated via individual interviews.

Nevertheless, it is imperative to acknowledge the existing ambiguity surrounding the relationship between non-coffee beverages—such as tea, cocoa/chocolate, and soft drinks—and their effects on NAFLD and liver fibrosis. Most of the current evidence stems from preclinical studies, while research focused on humans, especially those with metabolic syndrome, is relatively scarce. In response, our study also aimed to address this gap by focusing on the association of NAFLD and the consumption of non-coffee beverages in patients with metabolic syndrome. Our conclusions are based on an extensive compilation of data covering a range of beverages, incorporating detailed information on consumption patterns and caffeine levels.

Several limitations of our study warrant discussion. First, the sample size was not directly calculated based on our primary objective. Instead, the NAFLD population was derived from a cohort of patients with metabolic syndrome who were part of a larger project investigating the prevalence of NAFLD in patients at Siriraj Hospital, Thailand. The participants were randomly selected from the internal medicine outpatient department. Patients consented to complete a questionnaire and undergo ultrasonography. The total sample size of 505 patients was deemed sufficient to discern a difference in the NAFLD prevalence between those consuming ≥3 cups of coffee daily and those with lower consumption (less than 1 cup per day). Retrospectively calculating the sample size for this specific hypothesis test yielded an estimated power of approximately 80%. However, the relatively modest sample size constrained our ability to detect advanced liver fibrosis, as only 39 patients were diagnosed with advanced liver fibrosis. Future studies exploring the link between beverage consumption and liver fibrosis would benefit from a larger cohort.

Our investigation revealed no significant correlation between beverage consumption behaviors and NAFLD or advanced liver fibrosis prevalence. This outcome, however, was derived from a single questionnaire administered once, potentially introducing bias, particularly recall bias. Additionally, the questionnaire did not account for brewing methods or drink varieties, factors that could influence associations with NAFLD and its sequelae. The absence of pathological confirmation of the disease (due to ethical considerations against performing liver biopsies in this cohort) might also impact the results. Nonetheless, the ultrasonography and transient elastography methods employed in this study are valid non-invasive means of identifying NAFLD and liver fibrosis.

Furthermore, our study did not assess total daily caloric intake, a known confounding factor influencing NAFLD prevalence and its progression. Accurately quantifying total caloric intake was impeded by the heterogeneity of food types and the variability in daily consumption, thus representing a significant limitation within this research domain.

Given our study’s cross-sectional design, we cannot infer temporal or causal relationships between beverage consumption and the development of NAFLD or liver fibrosis. To address these limitations and establish causality, further research is imperative. This includes longitudinal studies, such as prospective cohort studies and rigorously designed randomized controlled trials, which could provide more definitive insights into the specific effects of different beverages on NAFLD prevalence. Despite these limitations, our study contributes valuable information to the existing body of knowledge, particularly in filling the research gap regarding beverage consumption and NAFLD among patients with metabolic syndrome in Thailand.

## 5 Conclusion

Our investigation revealed no marked differences in drinking behavior between patients with and without NAFLD. Our data did not reveal clear associations between the consumption of coffee, tea, cocoa/chocolate, or soft drinks and NAFLD or liver fibrosis in patients with metabolic syndrome. However, an exception was observed for high coffee consumption (three or more cups daily), which correlated with a reduced prevalence of NAFLD.

## Data availability statement

The raw data supporting the conclusions of this article will be made available by the authors, without undue reservation.

## Ethics statement

The studies involving humans were approved by the Siriraj Institutional Review Board. The studies were conducted in accordance with the local legislation and institutional requirements. The participants provided their written informed consent to participate in this study.

## Author contributions

CK: Conceptualization, Data curation, Formal analysis, Methodology, Visualization, Writing – original draft, Writing – review & editing. SN: Formal analysis, Visualization, Writing – original draft, Writing – review & editing. SS: Formal analysis, Visualization, Writing – original draft, Writing – review & editing. TC: Conceptualization, Data curation, Formal analysis, Investigation, Methodology, Resources, Supervision, Validation, Visualization, Writing – review & editing. WS: Data curation, Investigation, Validation, Writing – review & editing. CW: Data curation, Investigation, Validation, Writing – review & editing. CA: Data curation, Investigation, Validation, Writing – review & editing. TS: Data curation, Investigation, Validation, Writing – review & editing. RT: Data curation, Investigation, Validation, Writing – review & editing. NS: Data curation, Investigation, Validation, Writing – review & editing. PC: Conceptualization, Data curation, Resources, Validation, Writing – review & editing. PP: Conceptualization, Data curation, Formal analysis, Funding acquisition, Investigation, Methodology, Project administration, Resources, Supervision, Validation, Visualization, Writing – original draft, Writing – review & editing.

## References

[1] López-Velázquez JA Silva-Vidal KV Ponciano-Rodríguez G Chávez-Tapia NC Arrese M Uribe M. The prevalence of nonalcoholic fatty liver disease in the Americas. Ann Hepatol. (2014) 13:166–78. doi: 10.1016/S1665-2681(19)30879-8, PMID: 24552858

[2] Saklayen MG. The global epidemic of the metabolic syndrome. Curr Hypertens Rep. (2018) 20:12. doi: 10.1007/s11906-018-0812-z, PMID: 29480368 PMC5866840

[3] Chalasani N Younossi Z Lavine JE Charlton M Cusi K Rinella M. The diagnosis and management of nonalcoholic fatty liver disease: practice guidance from the American Association for the Study of Liver Diseases. Hepatology. (2018) 67:328–57. doi: 10.1002/hep.29367, PMID: 28714183

[4] Kuchay MS Martinez-Montoro JI Choudhary NS Fernandez-Garcia JC Ramos-Molina B. Non-alcoholic fatty liver disease in lean and non-obese individuals: current and future challenges. Biomedicine. (2021) 9:1–21. doi: 10.3390/biomedicines9101346, PMID: 34680463 PMC8533092

[5] Rinella ME Neuschwander-Tetri BA Siddiqui MS Abdelmalek MF Caldwell S Barb D. AASLD practice guidance on the clinical assessment and management of nonalcoholic fatty liver disease. Hepatology. (2023) 77:1797–835. doi: 10.1097/HEP.0000000000000323, PMID: 36727674 PMC10735173

[6] Cano-Marquina A Tarín JJ Cano A. The impact of coffee on health. Maturitas. (2013) 75:7–21. doi: 10.1016/j.maturitas.2013.02.002 23465359

[7] Higdon JV Frei B. Coffee and health: a review of recent human research. Crit Rev Food Sci Nutr. (2006) 46:101–23. doi: 10.1080/10408390500400009 16507475

[8] Saab S Mallam D Cox GA 2nd Tong MJ. Impact of coffee on liver diseases: a systematic review. Liver Int. (2014) 34:495–504. doi: 10.1111/liv.12304, PMID: 24102757

[9] Zhou J Ho C-T Long P Meng Q Zhang L Wan X. Preventive efficiency of Green tea and its components on nonalcoholic fatty liver disease. J Agric Food Chem. (2019) 67:5306–17. doi: 10.1021/acs.jafc.8b05032, PMID: 30892882

[10] Mahmoodi M Hosseini R Kazemi A Ofori-Asenso R Mazidi M Mazloomi SM. Effects of green tea or green tea catechin on liver enzymes in healthy individuals and people with nonalcoholic fatty liver disease: a systematic review and meta-analysis of randomized clinical trials. Phytother Res. (2020) 34:1587–98. doi: 10.1002/ptr.6637 32067271

[11] Tabatabaee SM Alavian SM Ghalichi L Miryounesi SM Mousavizadeh K Jazayeri S. Green tea in non-alcoholic fatty liver disease: a double blind randomized clinical trial. Hepat Mon. (2017) 17:e14993. doi: 10.5812/hepatmon.14993

[12] Shen H Rodriguez AC Shiani A Lipka S Shahzad G Kumar A. Association between caffeine consumption and nonalcoholic fatty liver disease: a systemic review and meta-analysis. Ther Adv Gastroenterol. (2016) 9:113–20. doi: 10.1177/1756283X15593700, PMID: 26770272 PMC4699270

[13] Alavinejad P Farsi F Rezazadeh A Mahmoodi M Eskandar H Masjedizadeh R. The effects of dark chocolate consumption on lipid profile, fasting blood sugar, liver enzymes, inflammation, and antioxidant status in patients with non-alcoholic fatty liver disease: a randomized, placebo-controlled, pilot study. J Gastroenterol Hepatol Res. (2015) 4:1858–64. doi: 10.17554/j.issn.2224-3992.2015.04.589

[14] Malhi H Loomba R. Editorial: dark chocolate may improve NAFLD and metabolic syndrome by reducing oxidative stress. Aliment Pharmacol Ther. (2016) 44:533–4. doi: 10.1111/apt.13716, PMID: 27484935 PMC4975527

[15] Loffredo L Baratta F Ludovica P Battaglia S Carnevale R Nocella C. Effects of dark chocolate on endothelial function in patients with non-alcoholic steatohepatitis. Nutr Metab Cardiovasc Dis. (2017) 28:143–9. doi: 10.1016/j.numecd.2017.10.027, PMID: 29329924

[16] Zhang Y Liu Z Choudhury T Cornelis MC Liu W. Habitual coffee intake and risk for nonalcoholic fatty liver disease: a two-sample Mendelian randomization study. Eur J Nutr. (2021) 60:1761–7. doi: 10.1007/s00394-020-02369-z, PMID: 32856188 PMC7910323

[17] Alberti KG Eckel RH Grundy SM Zimmet PZ Cleeman JI Donato KA. Harmonizing the metabolic syndrome: a joint interim statement of the international diabetes federation task force on epidemiology and prevention; National Heart, Lung, and Blood Institute; American Heart Association; World Heart Federation; International Atherosclerosis Society; and International Association for the Study of Obesity. Circulation. (2009) 120:1640–5. doi: 10.1161/CIRCULATIONAHA.109.192644, PMID: 19805654

[18] Rezaianzadeh A Namayandeh SM Sadr SM. National Cholesterol Education Program Adult Treatment Panel III versus international diabetic federation definition of metabolic syndrome, which one is associated with diabetes mellitus and coronary artery disease? Int J Prev Med. (2012) 3:552–8. PMID: 22973485 PMC3429802

[19] Chhimwal J Patial V Padwad Y. Beverages and non-alcoholic fatty liver disease (NAFLD): think before you drink. Clin Nutr. (2021) 40:2508–19. doi: 10.1016/j.clnu.2021.04.011, PMID: 33932796

[20] Jensen T Abdelmalek MF Sullivan S Nadeau KJ Green M Roncal C. Fructose and sugar: a major mediator of non-alcoholic fatty liver disease. J Hepatol. (2018) 68:1063–75. doi: 10.1016/j.jhep.2018.01.019, PMID: 29408694 PMC5893377

[21] Muriel P López-Sánchez P Ramos-Tovar E. Fructose and the liver. Int J Mol Sci. (2021) 22:1–22. doi: 10.3390/ijms22136969, PMID: 34203484 PMC8267750

[22] Sun Y Yu B Wang Y Wang B Tan X Lu Y. Associations of sugar-sweetened beverages, artificially sweetened beverages, and pure fruit juice with nonalcoholic fatty liver disease: cross-sectional and longitudinal study. Endocr Pract. (2023) 29:735–42. doi: 10.1016/j.eprac.2023.06.002, PMID: 37543090

[23] Arauz J Zarco N Segovia J Shibayama M Tsutsumi V Muriel P. Caffeine prevents experimental liver fibrosis by blocking the expression of TGF-β. Eur J Gastroenterol Hepatol. (2014) 26:164–73. doi: 10.1097/MEG.0b013e3283644e26, PMID: 23903851

[24] Furtado KS Prado MG Aguiar ESMA Dias MC Rivelli DP Rodrigues MA. Coffee and caffeine protect against liver injury induced by thioacetamide in male Wistar rats. Basic Clin Pharmacol Toxicol. (2012) 111:339–47. doi: 10.1111/j.1742-7843.2012.00903.x, PMID: 22646289

[25] Shim SG Jun DW Kim EK Saeed WK Lee KN Lee HL. Caffeine attenuates liver fibrosis via defective adhesion of hepatic stellate cells in cirrhotic model. J Gastroenterol Hepatol. (2013) 28:1877–84. doi: 10.1111/jgh.12317, PMID: 23808892

[26] Chen S Teoh NC Chitturi S Farrell GC. Coffee and non-alcoholic fatty liver disease: brewing evidence for hepatoprotection? J Gastroenterol Hepatol. (2014) 29:435–41. doi: 10.1111/jgh.12422, PMID: 24199670

[27] Birerdinc A Stepanova M Pawloski L Younossi ZM. Caffeine is protective in patients with non-alcoholic fatty liver disease. Aliment Pharmacol Ther. (2012) 35:76–82. doi: 10.1111/j.1365-2036.2011.04916.x 22059453

[28] Marventano S Salomone F Godos J Pluchinotta F Del Rio D Mistretta A. Coffee and tea consumption in relation with non-alcoholic fatty liver and metabolic syndrome: a systematic review and meta-analysis of observational studies. Clin Nutr. (2016) 35:1269–81. doi: 10.1016/j.clnu.2016.03.012 27060021

[29] Ebadi M Ip S Bhanji RA Montano-Loza AJ. Effect of coffee consumption on non-alcoholic fatty liver disease incidence, prevalence and risk of significant liver fibrosis: systematic review with Meta-analysis of observational studies. Nutrients. (2021) 13:1–16. doi: 10.3390/nu13093042 PMC8471033 34578919

[30] Chen YP Lu FB Hu YB Xu LM Zheng MH Hu ED. A systematic review and a dose-response meta-analysis of coffee dose and nonalcoholic fatty liver disease. Clin Nutr. (2019) 38:2552–7. doi: 10.1016/j.clnu.2018.11.030, PMID: 30573353

[31] Wijarnpreecha K Thongprayoon C Ungprasert P. Coffee consumption and risk of nonalcoholic fatty liver disease: a systematic review and meta-analysis. Eur J Gastroenterol Hepatol. (2017) 29:e8–e12. doi: 10.1097/MEG.0000000000000776, PMID: 27824642

