# Supplementary material for: Beverage consumption in patients with metabolic syndrome and its association with non-alcoholic fatty liver disease: a cross-sectional study
Source: Front Nutr. 2024 Jan 22;11:1257969. doi: 10.3389/fnut.2024.1257969 (PMC10839077; doi:10.3389/fnut.2024.1257969)
Supplement: Supplementary file 1 [file Data_Sheet_1.PDF]

Supplementary Material

Beverage consumption in patients with metabolic syndrome and its association with nonalcoholic fatty liver disease: a cross-sectional study

Chayanis Kositamongkol[1,2], Sorawis Ngaohirunpat[3], Supawit Samchusri[3], Thanet Chaisathaphol[1], Weerachai Srivanichakorn[1], Chaiwat Washirasaksiri[1], Chonticha Auesomwang[1], Tullaya Sitasuwan[1], Rungsima Tinmanee[1], Naruemit Sayabovorn[1], Phunchai Charatcharoenwitthaya[4], and Pochamana Phisalprapa[1]*

[1]Division of Ambulatory Medicine, Department of Medicine, Faculty of Medicine Siriraj Hospital, Mahidol University, Bangkok, Thailand

[2]Department of Clinical Epidemiology, Faculty of Medicine, Thammasat University, Pathumthani, Thailand

[3]Faculty of Medicine Siriraj Hospital, Mahidol University, Bangkok, Thailand

[4]Division of Gastroenterology, Department of Medicine, Faculty of Medicine Siriraj Hospital, Mahidol University, Bangkok, Thailand

***Correspondence:**
Pochamana Phisalprapa, MD, PhD
Email: coco_a105@hotmail.com; Tel./Fax: +66 2 419 7190

Keywords: caffeine, cocoa, coffee, fibrosis, metabolic syndrome, nonalcoholic fatty liver disease, steatosis, tea

Supplementary Material 1 Beverage Consumption Behavior Questionnaire (English-translation)

Patient's Code: ________ Date: ___/___/___

Coffee:

1. Have you ever had coffee? ☐ Yes ☐ No
2. Do you drink coffee now? ☐ Yes ☐ No
3. How long since you stopped drinking coffee? ___Years ___Months ___Weeks ___Days
4. What kind of coffee do you usually have? (Check the picture chart)
   4.1 ____________ 4.2 ____________ 4.3 ____________
5. How much coffee do you have each day? (Please circle the unit)
   5.1 _____ pack/cup/can/bottle per day 5.2_____ days per week/month
6. Do you add anything to your coffee? Sugar: ___ teaspoon, Sweetener: ___ teaspoon, Creamer: ___ teaspoon, Condensed milk: ___ teaspoon

Tea:

1. Have you ever had tea? ☐ Yes ☐ No
2. Do you drink tea now? ☐ Yes ☐ No
3. How long since you stopped drinking tea? ___Years ___Months ___Weeks ___Days
4. What kind of tea do you usually have? (Check the picture chart)
   4.1 ____________ 4.2 ____________ 4.3 ____________
5. How much tea do you have each day?
   5.1 _____ pack/cup/can/bottle per day 5.2_____ days per week/month
7. Do you add anything to your tea? Sugar: ___ teaspoon, Sweetener: ___ teaspoon, Creamer: ___ teaspoon, Condensed milk: ___ teaspoon

Cocoa/chocolate:

1. Have you ever had cocoa/chocolate? ☐ Yes ☐ No
2. Do you drink cocoa/chocolate now? ☐ Yes ☐ No
3. How long since you stopped drinking cocoa/chocolate? ___Years ___Months ___Weeks ___Days
4. What kind of cocoa/chocolate do you usually have? (Check the picture chart)
   4.1 ____________ 4.2 ____________ 4.3 ____________
5. How much cocoa/chocolate do you have each day?
   5.1 _____ pack/cup/can/bottle per day 5.2_____ days per week/month
6. Do you add anything to your cocoa/chocolate? Sugar: ___ teaspoon, Sweetener: ___ teaspoon, Creamer: ___ teaspoon, Condensed milk: ___ teaspoon

Soft drinks:

1. Have you ever had soft drinks? ☐ Yes ☐ No
2. Do you drink soft drinks now? ☐ Yes ☐ No
3. How long since you stopped drinking soft drinks? ___Years ___Months ___Weeks ___Days
4. What kind of soft drinks do you usually have? (Check the picture chart)
   4.1 ____________ 4.2 ____________ 4.3 ____________
5. How much soft drinks do you have each day?
   5.1 _____ pack/cup/can/bottle per day 5.2_____ days per week/month

Supplementary Tables

Supplementary Table 1 Caffeine and energy data listed in product nutrition facts labels

Products	Caffeine (mg/100 ml)	Energy (kcal/100 ml)	Volume factor (100 ml/unit)	Caffeine per unit (mg)	Energy per unit (kcal)
**Coffee**					
Arabus: Caramel Macchiato	39.2	75	2.0	78.4	150
Arabus: Espresso cup	45.3	46.7	2.0	90.6	93.3
Arabus: Latte cup	45.4	65	2.0	90.8	130
Arabus: Mocha cup	48.5	75	2.0	97	150
Arabus: Other	44.6	65.4	2.0	89.2	130.8
Birdy: 3 in 1	22.8	28	2.5	57	70
Birdy: 3 in 1 (Green)	22.8	24	2.5	57	60
Birdy: 3 in 1 (Red)	22.8	28	2.5	57	70
Birdy: 3 in 1 (Yellow)	22.8	32	2.5	57	80
Birdy: Barista Espresso Shot can (Brown)	75	46.7	1.5	112.5	70
Birdy: Black can	85	16.7	1.8	153	30
Birdy: Black Low Sugar Formula can (Black)	75	19.4	1.8	135	35
Birdy: Green can	73	38.9	1.8	131.4	70
Birdy: Latte can (Brown)	48	50	1.8	86.4	90
Birdy: Red	51.9	30.7	2.2	111.6	65
Birdy: Robusta can (Red)	81	33.3	1.8	145.8	60
Birdy: Robusta Low Sugar Formula can (Orangish-Brown)	67	25	1.8	120.6	45
Birdy: Other	57.2	31.4	2	113.3	61
Birdy: Other can	72	32.9	1.8	126.5	57.1
Equal Café Cappuccino	22.8	24	2.5	57	60
Freshly brewed coffee / Thai traditional coffee / Hot Vietnamese coffee	39.4	65.6	1.8	71	118
Ginseng/Lingzhi coffee	39.4	50	1.8	71	90
Healthy coffee	22.8	22	2.5	57	55
Iced coffee	28.4	126.8	2.5	71	317
Khao Shong: 3 in 1	22.8	35.2	2.5	57	88
Khao Shong: Cappuccino	22.8	36	2.5	57	90
Khao Shong: Condensed Milk Flavour	22.8	36	2.5	57	90
Khao Shong: Espresso	22.8	28	2.5	57	70
Khao Shong: Mocha	22.8	40	2.5	57	100
Khao Shong: Super rich	22.8	36	2.5	57	90
Meiji: Coffee flavored milk	0	90	2.0	0	180
Moccona Trio 3 in 1: Cappuccino	22.8	28	2.5	57	70
Moccona Trio 3 in 1: Classic	22.8	32	2.5	57	80
Moccona Trio 3 in 1: Espresso	22.8	32	2.5	57	80
Moccona Trio 3 in 1: Other	22.8	30.7	2.5	57	76.7
Naturegift coffee	22.8	24	2.5	57	60

Products	Caffeine (mg/100 ml)	Energy (kcal/100 ml)	Volume factor (100 ml/unit)	Caffeine per unit (mg)	Energy per unit (kcal)
Nescafe 3 in 1: Americano	22.8	14	2.5	57	35
Nescafe 3 in 1: Espresso Roast (Green)	22.8	28	2.5	57	70
Nescafe 3 in 1: Other	22.8	24.7	2.5	57	61.7
Nescafe 3 in 1: Red/Green	22.8	30	2.5	57	75
Nescafe 3 in 1: Rich Aroma	22.8	32	2.5	57	80
Nescafe Gold 2 in 1: No sugar	22.8	20	2.5	57	50
Nescafe Protect Proslim	22.8	24	2.5	57	60
Nescafe Red Cup: No sugar	22.8	2	2.5	57	5
Nescafe: Cappuccino can	70	55.6	1.8	126	100
Nescafe: Espresso Roast can	80	38.9	1.8	144	70
Nescafe: Other	35.9	26.8	2.3	83.4	58.8
Nescafe: Other can	75	47.2	1.8	135	85
Other 2 in 1 coffee	22.8	20	2.5	57	50
Other 3 in 1 coffee	22.8	27.8	2.5	57	69.4
Other black coffee	22.8	2	2.5	57	5
Other canned coffee	71.3	34.7	1.8	126.2	61
Preaw coffee: can	59	22.2	1.8	106.2	40
Preaw coffee: Other	22.8	18	2.5	57	45
Super coffee	22.8	36	2.5	57	90
Thai style black coffee	28.4	66	2.5	71	165
Truslen	22.8	22	2.5	57	55
Truslen: Coffee Bloc	22.8	20	2.5	57	50
Truslen: Coffee Plus Bern	22.8	20	2.5	57	50
Truslen: Coffee Plus Collagen	22.8	24	2.5	57	60
Truslen: Coffee Plus Sugar free	22.8	24	2.5	57	60
**Tea**					
Fuji Green Tea (bottle)	7.9	12.4	5	39.6	61.9
Fuji Green Tea (bottle, green cap, sugar)	8.4	20.8	5	41.8	103.8
Fuji Green Tea (bottle, white lid, no sugar)	7.5	4	5	37.5	20
Ginseng Tea	11	0.8	2.5	27.5	2
Homemade Chinese Tea	14.8	0.8	2.5	37	2
Homemade Green Tea / Herbalite Green Tea	10	14.4	2.5	25	36
Iced Black Tea	16.8	44	2.5	42	110
Iced Tea / Thai Traditional Tea	16.8	128	2.5	42	320
Ichitan Bottle Honey Lemon Flavor	10.4	38.1	4.2	43.7	160
Ichitan Bottle Japanese Rice Germ	8.9	16.7	4.2	37.4	70
Ichitan Bottle Original Flavor	8.8	14.3	4.2	37	60
Ichitan Honey Lemon	8.8	52.4	4.2	37.1	220
Ichitan Honey Lemon Flavor	9.6	45.2	4.2	40.4	190

Products	Caffeine (mg/100 ml)	Energy (kcal/100 ml)	Volume factor (100 ml/unit)	Caffeine per unit (mg)	Energy per unit (kcal)
Ichitan Japanese Rice	8.1	47.4	3.8	30.8	180
Lemon Tea	16.8	86	2.5	42	215
Lipton Ice Tea (bottle)	10	39.6	4.8	48	190
Lipton Ice Tea (can)	9	46.2	3.3	29.3	150
Lipton Ice Tea: Peach (bottle)	4.4	50	2	8.8	100
Lipton Tea	7.9	33.9	3.1	24.6	110
Lipton Tea (no sugar)	8	0	2.5	20	0
Mulberry / Safflower / Lemongrass / Banquet / Fruit Tea	0	9.8	2.5	0	24.5
Nature Gift White Tea	11.2	0.8	2.5	28	2
Nestea	5.2	36	2.5	13	90
Oishi Gold Bottle Genmaicha (Delight Formula)	3.4	15	4	13.5	60
Oishi Gold Bottle Genmaicha (No Sugar Formula)	3	0	4	11.8	0
Oishi Gold Kabusecha Bottle (No Sugar Formula)	9.2	0	4	36.8	0
Oishi Green Tea Bottle Apple Honey Flavor (No Sugar Formula) (380 ml)	5	0	3.8	18.8	0
Oishi Green Tea Bottle Honey Lemon Flavor (500 ml)	12.9	47.4	3.8	49	180
Oishi Green Tea Bottle Kyoho Grape Flavor mixed with coconut jelly (380 ml)	4.6	36.8	3.8	17.3	140
Oishi green tea bottle: Honey lemon	10.5	26.3	3.8	40	100
Oishi green tea bottle: Original (380 ml)	8.1	23.7	3.8	30.6	90
Oishi green tea bottle: Original (500 ml)	8.2	24	5	41.1	120
Oishi green tea carton: Honey lemon	11.1	47.2	2.5	27.8	118
Oishi Green Tea Honey Lime Flavor Bottle Double Lemon Formula (380 ml)	7.4	40	5	36.8	200
Oishi Green Tea Watermelon Bottle (380 ml)	4.9	36.8	3.8	18.6	140
Oishi Green Tea: Japanese Rice Flavor Bottle (500 ml)	8.9	18	5	44.4	90
Oishi Honey	10.5	40.2	3.8	39.5	149.5
Oishi Japanese Rice	9.2	37.5	3.8	34.7	129.3
Oishi Japanese Rice (Bottle)	10.1	47.4	3.8	38.5	180
Oishi Japanese Rice (Carton)	8.6	47.2	2.5	21.5	118
Oishi Tea	7.7	27.3	3.9	30.1	102.4

Products	Caffeine (mg/100 ml)	Energy (kcal/100 ml)	Volume factor (100 ml/unit)	Caffeine per unit (mg)	Energy per unit (kcal)
Other bottled tea	6.8	27	4	27.3	106.1
Other green tea	8	27.4	4.1	32.5	106.8
Puriku White Tea: Honey Lemon	12.1	31.4	3.5	42.4	110
Puriku White Tea: Mixed Berry	0	25.7	3.5	0	90
Puriku White: Chrysanthemum	0	22.9	3.5	0	80
Puriku: Pomegranate	0	22.9	3.5	0	80
Puriku: Strawberry	0	28.6	3.5	0	100
**Chocolate / Cocoa**					
Cocoa	2.0	75.4	2.5	5.0	188.5
Chocolate 3 in 1: Other	0.0	82.5	2.0	0.0	165.0
Dutch Mill: Chocolate Milk	0.0	85.0	2.0	0.0	170.0
Foremost: Chocolate Milk	0.0	91.9	1.9	0.0	170.0
Meiji: Chocolate Milk	0.0	65.0	2.0	0.0	130.0
Milo	0.0	20.0	2.0	0.0	40.0
Milo 3 in 1: box	0.0	65.0	2.0	0.0	130.0
Ovaltine	0.0	20.0	2.0	0.0	40.0
Ovaltine 3 in 1: box	0.0	100.0	2.0	0.0	200.0
Other chocolate milk	0.0	80.6	2.0	0.0	156.7
Other cocoa	0.8	66.1	2.3	2.0	155.5
Other iced chocolate	2.0	48.0	2.5	5.0	120.0
Starbuck Hot Cocoa	5.2	74.4	4.8	25.0	357.0
**Soft drinks**					
7-up	0	54.3	3.7	0	200
Bailey's Orange Juice	0	35.9	2.9	0	105
Big: Orange Flavor	0	41.9	4.7	0	195
Coke (350 ml)	9.7	23.8	3.5	33.8	74.3
Coke (370 ml)	9.2	38	3.7	34	140
Coke original (250 ml)	9.2	44	2.5	23.1	110
Coke original (310 ml)	9.2	41.7	3.1	29	130
Coke original (325 ml)	9.2	43.1	3.3	30	140
Coke zero (325 ml)	9.2	0	3.3	30	0
Coke zero (400 ml)	10.2	0	4	40.7	0
Coke zero (500 ml)	9.2	0	5	46.2	0
Coke zero / Diet coke (370 ml)	12.2	0	3.7	45	0
Est (250 ml)	9.8	40	2.5	24.5	100
Est (400 ml)	12.1	23.1	4	49	89.6
Est (515 ml)	9.8	40	5	48.9	200
Fanta	0	53	3.7	0	195
Fanta Mixed	0	34.1	3.4	0	118.3
Fanta: Orange Flavor	0	18.5	3.3	0	60
Fanta: Strawberry Flavor	0	30.8	3.3	0	100
Minute Maid	0	35.9	2.9	0	105
Minute Maid Pulpy	0	35.8	3.4	0	120
Minute Maid Splash	0	36	2.5	0	90

Products	Caffeine (mg/100 ml)	Energy (kcal/100 ml)	Volume factor (100 ml/unit)	Caffeine per unit (mg)	Energy per unit (kcal)
Mirinda: Orange Flavor	0	29	14.5	0	420
Other orange juice	0	30	5.3	0	159.2
Other soda	5.5	29.5	4	21.8	118.7
Pepsi (370 ml)	10.3	40.8	3.7	38	150
Pepsi (460 ml)	14.5	22.5	4.6	66.7	105
Pepsi (550 ml)	10.3	49.1	5.5	56.8	270
Pepsi Max	18.8	0	3.7	69	0
Pepsi Max	18.8	0	4.6	86.1	0
Pepsi Max Taste (550 ml)	18.8	0	5.5	103.1	0
Pepsi original	10.3	44.9	4.6	47.4	210
RC Cola	12.1	23.1	4	49	89.6
Schweppes	0	46.2	3.7	0	170
Splash	0	19	3.7	0	70
Splash Mixed	0	27.5	3.1	0	80
Sprite	0	54.3	3.7	0	200
Sprite Mixed	0	39.5	3.5	0	140
